# How nursing students develop resilience through self-compassion and self-leadership: a mixed methods study

**DOI:** 10.1016/j.ijnsa.2026.100693

**Published:** 2026-10-01

**Authors:** Heleen C. Melissant, Claudia M. Hernandez Murillo, Kerry A. Sherman, Monique F. Crane, Rick R.A. Hendriks, Kim J. Verhaegh

**Affiliations:** a Leiden University of Applied Sciences, Department of Health, Zernikedreef 11, Postbus 382, Leiden, 2300 AJ, the Netherlands; b Lifespan Health and Wellbeing Research Centre, Macquarie University, Sydney, Australia; c School of Psychological Sciences, Macquarie University, Sydney, Australia; d Performance and Expertise Research Centre, School of Psychological Sciences, Macquarie University, Sydney, Australia; e Alrijne Hospital, Department of Alrijne Academy, Simon Smitweg 1, Leiderdorp, 2353 GA, the Netherlands

**Keywords:** Self-compassion, Students, Nursing, Leadership, Resilience, Psychological, Vitality, Education, Nursing, Internship and Residency

## Abstract

**Background:**

Nursing students often experience high levels of stress due to various challenges during internships. To manage these challenges, students need to develop strategies, such as self-leadership and self-compassion. While the effectiveness of these factors has been explored, little research has investigated how students use them to remain resilient in adverse circumstances.

**Objectives:**

This exploratory mixed-methods study combining qualitative and quantitative research aimed to examine how nursing students apply self-compassion and self-leadership to foster resilience. Qualitative data collected through 15 semi-structured interviews, were analyzed using reflexive thematic analysis. Quantitative data were collected using the Self-Compassion Scale – Short Form, the Abbreviated Self-leadership Questionnaire, and the Vita-16. While the qualitative component focused specifically on resilience, the quantitative component examined vitality as a broader indicator of resilience. Participants were Bachelor of Nursing students in the later stages of their program, enrolled in one of three educational pathways: full-time (primarily students entering from secondary education), dual (students combining study with employment in healthcare) and modular (flexible programs for lateral entrants, often with prior healthcare experience such as vocationally trained nurses or professionals in related fields). All participants had substantial internship experience.

**Results:**

The qualitative analysis identified three main themes: (1) connecting with one's inner nurse, (2) being a brave nursing student, and (3) building strength through self-compassion. Students reported learning to apply these strategies amidst high pressure working circumstances. A total of 137 students participated in the quantitative research (87% female; mean age 24, SD = 6.6). Hierarchical regression analyses revealed that self-compassion (B = 0.47, 95% CI [0.34, 0.61], p < .001) and self-leadership (B = 0.66, 95% CI [0.45, 0.87], p < .001) were significant positive predictors of vitality. The final model explained 54.0% of the variance in vitality, whereas sociodemographic characteristics did not contribute significantly.

**Conclusion:**

Self-leadership and self-compassion are crucial skills in fostering resilience among nursing students. Educational programs and clinical practices should take initiatives to promote these skills while also taking proactive steps to protect students by creating safe environments.

**What is already known**

• Nursing students are confronted with adverse circumstances during internships in a high pressure healthcare environment
• Building resilience is crucial to effectively manage these challenges
• Self-compassion and self-leadership strategies can be helpful to build resilience in nursing students

**What this paper adds**

• Insight into how self-compassion and self-leadership strategies are built and applied during internships
• Insight into how connecting with their 'inner nurse' helps students to stay motivated
• An explanation of how nursing students build strength through being brave and self-compassionate

## 1 Introduction

Nursing education is a challenging experience where nursing students are expected to successfully perform clinical rotations, exams, and demanding coursework. These challenges are compounded by the realities of working in a demanding healthcare system under pressure, which can impact their learning experiences, such as an absence of supervision or a high workload and responsibilities (Kox et al., 2020; Canzan et al., 2022).

As healthcare systems in the Netherlands and around the world face increasing pressure, it becomes essential to support nursing students in developing the skills necessary to cope with difficult circumstances and safeguard their well-being. Resilience can be understood as a dynamic and ongoing process that enables nurses to adapt positively to workplace stressors, maintain well-being, and continue delivering safe, high quality patient care. This requires both individual resources and supportive organizational conditions (Cooper et al., 2020). Resilience is considered a key dimension of vitality, a broader construct that refers to feeling energetic, motivated and capable of coping with stressors (Strijk, 2015).

Traynor's critical perspective on resilience (Traynor, 2017) argues that resilience should not be viewed solely as an individual capacity to adapt to adversity. Rather, structural and organizational factors influence the challenges individual nurses face, as well as their ability to respond to them. In line with this perspective, this study recognizes resilience as a result of an interaction between personal and contextual factors. However, the focus of this study is on two personal resources that may support resilience in nursing students: self-compassion and self-leadership.

A personal resource that can enhance resilience is self-compassion, referring to the ability to treat oneself with kindness, understanding, and acceptance when faced with failures and setbacks (Neff, 2011). Research has shown that emerging adults with high levels of self-compassion have lower levels of depressive symptoms and greater life satisfaction (Fenzel and Richardson, 2022). For nursing students specifically, self-compassion and resilience are associated with improved mental health and well-being (Kotera et al., 2021).

Another essential personal resource is the use of self-leadership. Self-leadership refers to a set of cognitive and behavioral strategies individuals use to enhance their personal effectiveness and performance. Setting personal goals, visualizing successful performance, evaluating one’s beliefs and assumptions, providing self-rewards and engaging in constructive self-talk characterize self-leadership (Houghton, 2012). By cultivating these practices, nursing students may strengthen their resilience and their capacity to navigate the demands of their education and future professional roles (Pursio et al., 2025).

Nursing students who exhibit higher levels of self-compassion, self-leadership, and resilience experience lower levels of stress and burnout and higher levels of job satisfaction and academic performance (Gilmartin et al., 2017; Hughes et al., 2021). To clarify the relationship between these concepts, this study draws on the Study Demands-Resources (SD-R) framework (Bakker and Mostert, 2024) in which personal resources play an important role in coping with study demands. Within this framework, self-compassion and self-leadership are viewed as complementary personal resources. Self-compassion reflects emotional regulation by enabling students to respond to setbacks with self-kindness, whereas self-leadership reflects proactive behavioral and cognitive self-regulation strategies such as goal-setting and self-observation. Resilience is seen as an adaptive capacity that may be supported by the use of self-compassion and self-leadership, to help nursing students to overcome challenges during their internships (Fig. 1).

**Fig. 1:**
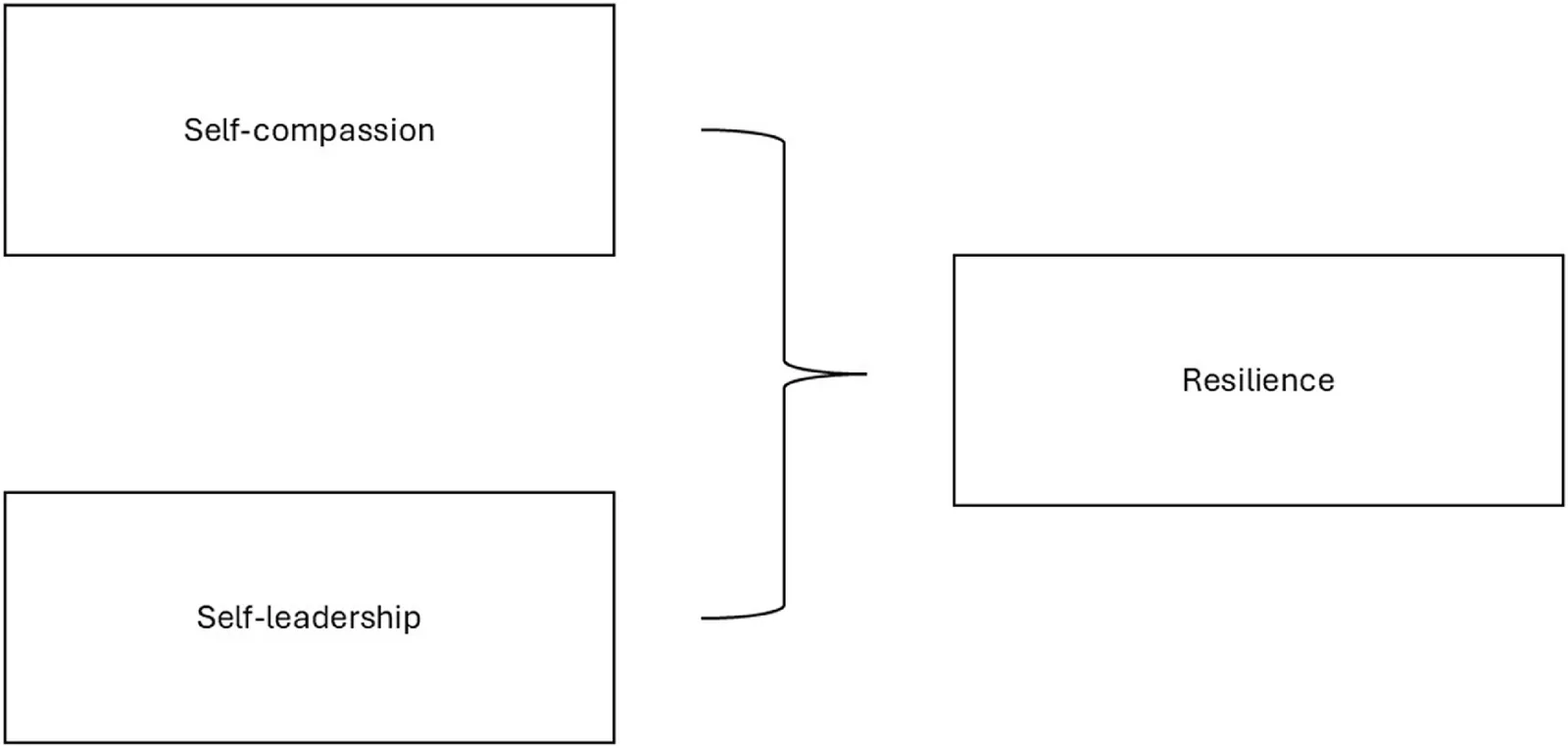
Conceptual model of resilience in nursing students.

Interventions targeting self-compassion and self-leadership can be effective in promoting well-being and performance in nursing students. In one study, self-compassion and self-leadership strategies formed the basis of a 10-week intervention for a student population (Sampl et al., 2017). The self-compassion component of the intervention included exercises on mindfulness, acceptance and attention, whereas the self-leadership component provided behavioral strategies (such as goal setting, self-reward and self-cueing), constructive thought strategies (such as visualizing successful performances) and natural reward strategies (creating and upholding motivation). Compared with the control group, students in the intervention group had better academic performance and lower stress levels in that semester.

Attention to self-compassion, self-leadership and resilience within nursing education is growing. Previous research has demonstrated positive associations between self-compassion and resilience among nursing students (Kotera et al., 2021). In contrast, self-leadership has mainly been studied in relation to autonomy and professional development (Scammell et al., 2020). Research on these concepts among nursing students has predominantly employed cross-sectional quantitative designs. To our knowledge, no studies have used a mixed-methods approach to examine resilience, self-compassion, and self-leadership together within the context of internships. Also, limited research has investigated how nursing students actually apply these resources when facing setbacks in clinical practice. Therefore, a mixed-methods design was considered appropriate to gain quantitative insight into the relationships between these constructs and qualitative insight into how students apply them during their internships.

Resilience is the primary concept of interest in this study. A mixed methods approach was considered appropriate because it allowed us to investigate resilience from complementary perspectives. The qualitative component explored how nursing students use self-compassion and self-leadership to build resilience during internships, whereas the quantitative component examined vitality, a broader construct that includes resilience as one of its key dimensions, in relation to self-compassion and self-leadership. Exploring these concepts from the perspective of nursing students is crucial for understanding how to create a supportive educational environment and inspire strategies to enhance learning and resilience.

Therefore, the aim of this mixed-methods study is to investigate how nursing students apply self-compassion and self-leadership skills to overcome adversities and build strength from them.

## 2 Methodology

### 2.1 Design, setting and participants

A convergent mixed-methods design was employed (Dawadi et al., 2021). An online quantitative survey and qualitative semi-structured interviews were conducted in parallel. The qualitative part was prioritized, because the primary aim of the study was to understand how students apply self-compassion and self-leadership in practice. Qualitative data were therefore considered essential for capturing the depth and complexity of these experiences, while quantitative data complemented and contextualized the qualitative findings. The study was reported in accordance with the Guidelines for Reporting Mixed Methods Research (GRAMMS) recommendations (O'Cathain et al., 2008).

The study was conducted among nursing students at the Leiden University of Applied Sciences. Data was collected between November 2022 and April 2023. Students in the later stages of the Bachelor of Nursing program were eligible to participate, since these students have experience with clinical placements and associated challenges. Eligible participants were third- and fourth-year students from the full-time and dual pathways and modular pathway students with clinical placement experience, enrolled in the Bachelor of Nursing program of Leiden University of Applied Sciences. The modular pathway is designed for lateral entrants with prior healthcare or professional experience and involves flexible, often shortened, trajectories. Students who could not speak or write Dutch were excluded from participation.

### 2.2 Qualitative study: recruitment, data generation and data analysis

For the qualitative study, the eligible population consisted of 614 third- and fourth-year nursing students enrolled in the full-time and dual pathways of the Bachelor of Nursing program. Within this population, participants were recruited in three ways: (Kox et al., 2020) lecturers, who were not part of the research team, promoted the study among third- and fourth year nursing students during classes and provided contact details of students who expressed interest. Those students were then approached by one of the researchers (CHM) via e-mail; (Canzan et al., 2022) Eligible students who were acquainted with the researcher who conducted the interviews (CHM) were approached to participate in the study; and, (Cooper et al., 2020) students who were enrolled in the Minor Nursing Leadership program were approached by a student assistant.

Semi-structured interviews were conducted. A topic list was developed encompassing five primary dimensions for exploration: (Kox et al., 2020) adversities and their impact; (Canzan et al., 2022) coping mechanisms applied during adversity; (Cooper et al., 2020) utilization of self-compassion skills; (Strijk, 2015) utilization of self-leadership skills; and (Traynor, 2017) needs and preferences to learn to deal with setbacks. In the development of this topic list, definitions from established sources for self-compassion, resilience, and self-leadership were adopted (Strijk, 2015; Neff, 2011; Houghton, 2012; Drenkard, 2012). After the first five interviews, the topic list was refined (Supplementary File I). The process was iterative, involving the adaptation of the topic list after conducting one or two interviews, continuing this cycle until the topic list was aligned with the intended study objective (Kallio et al., 2016). Interviews were conducted in a classroom at the university, during a regular study day, and were recorded with an audio recorder. The interviews lasted between 30–60 min. Ten of the interviews were conducted by CHM. CHM declares having no specific relationship with any of the participating students, nor did the students hold a dependent educational position towards the researcher. The other five interviews were conducted by a student assistant who was their peer. Due to the student assistant's role and relative inexperience with interviewing, these interviews were secondary to the other interviews. The researchers found the interview data sufficiently rich to construct a meaningful narrative. Prior to the analysis, participants received a summary of their interview to verify accuracy. No inaccuracy was reported.

The interviews were transcribed verbatim and analyzed using ATLAS.ti 23.2.1.26990 for Windows (Scientific Software Development GmbH, 2023). The phases of reflexive thematic analysis, as described by Braun and Clarke (Braun and Clarke, 2006; Braun and Clarke, 2022), were followed for analyzing the interviews. Data were analyzed following a constructivist approach, which assumes, according to Braun and Clarke (Braun and Clarke, 2022), that our reality is socially constructed and contextually situated. Two researchers performed the analysis. CHM, MSc in Nursing and RN, is lecturer in the Bachelor of Nursing program, primarily teaching first-year students. For her master thesis, she conducted a qualitative case study about providing person-centered dementia care. CHM participated in a research group for lecturers to improve and develop education. HCM, MSc in Health Psychology, obtained her PhD studying body image and sexuality in head and neck cancer patients. She evaluated a self-compassion intervention and conducted multiple qualitative studies. She teaches third- and fourth-year nursing students. Both researchers are members of the research group Nursing Leadership.

The two researchers independently coded the first interview and then reflected on the meaning of these codes. Based on these initial codes, the next interview was analyzed. After three interviews, CHM and HCM discussed the initial codes and developed preliminary themes, which were used as a basis for coding the subsequent interviews. This process was repeated until all interviews were analyzed. At last, the researchers discussed the meaning of the resulting themes, reconsidered them and defined their final meaning. During the analysis, a reflexive journal was maintained to document decisions, considerations, and agreements made in developing codes and themes. Also, a consultation with a researcher familiar with reflexive thematic analysis was held to provide feedback on the analysis process. In case of disagreement between the two researchers concerning the meaning of a theme, a third researcher was consulted (KV). This manuscript reports the methods and results of this qualitative study in accordance with the Reflexive Thematic Analysis Reporting Guidelines (RTARG) (Braun and Clarke, 2024).

### 2.3 Quantitative study: sample, data collection and data analysis

Students meeting the eligibility criteria described in section 2.1 (n = 873) were invited by one of the researchers (HCM) via e-mail to participate in the online survey on vitality, self-compassion, and self-leadership. A reminder was sent two months later. To maximize the response rate, participants had the chance to win a small incentive. Of these students, 137 completed the survey, resulting in a response rate of 16%.

The survey consisted of sociodemographic questions and measurement instruments on vitality, self-compassion, and self-leadership. The 12-item Self-Compassion Scale – Short Form (SCS-SF) was used to measure students’ self-compassion (Raes et al., 2011). Higher scores (mean range 1–7) indicate that students are more self-compassionate. The SCS-SF has adequate psychometric properties in past research (α ≥ 0,86 in all samples) and is translated and validated in Dutch (Raes et al., 2011, Neff and Vonk, 2009). In the current study internal consistency was good (α = 0,85).

The Vita-16 was selected as a validated Dutch instrument that conceptualizes resilience as one dimension of the broader construct of vitality (Strijk, 2015). At the outset of the study, the resilience dimension was considered relevant to the study objectives. However, exploratory factor analyses indicated that the proposed three-factor structure (energy, motivation, and resilience) was not supported in our sample. Consequently, only the total vitality score was used in the quantitative analyses. Previous validation studies have reported excellent reliability for the Vita-16 (α = 0.89–0.95) (Strijk, 2015), which was confirmed in the current study (α = 0.90). Higher scores (mean range 1–7) indicate higher perceived vitality.

And last, self-leadership was measured with the validated 9-item Abbreviated Self-Leadership Questionnaire (ASLQ) (Houghton, 2012). Past research shows good internal consistency (α = 0.73), and the current study found a similar level (α = 0.74). This instrument measures three dimensions of self-leadership strategies. A higher score (mean range 1–5) indicates higher self-leadership. For the current study, the English version of the ASLQ was translated in Dutch by a researcher (HCM) and optimized by a Dutch-speaking native English speaker. The total survey was delivered on the software program Easion v.3.129 and was pretested among four researchers before being finalized.

Statistical analyses were conducted in SPSS (IBM, United States of America, version 29.0.2.0). Descriptive statistics were generated to summarize participant sociodemographic characteristics. Total and mean scores were calculated for the self-compassion, self-leadership, and vitality variables. Statistical assumptions (normality, homoscedasticity and linearity) were checked. Following confirmation of normality, Pearson correlations were used to analyze the relationships among variables. A correlational threshold of 0.7 applied, in line with published guidance (Rovetta, 2020). A hierarchical regression analysis was conducted to investigate whether self-compassion and self-leadership can predict vitality and to what extent they contribute. In the first step, sociodemographic factors were entered as control variables. In the second step, self-compassion was added, followed by self-leadership in the third step. This approach allowed for the assessment of the incremental variance explained (ΔR²) by each construct. Additionally, coefficients of determination (R^2^), unstandardized coefficients (B), standard error (SE B), standardized coefficients (*β*), t-values, *F-*statistics, F-change (Δ*F*) and confidence intervals were calculated.

Independent variables included self-compassion, self-leadership and sociodemographic factors: gender (male vs. female, age (18–21 vs. 22–55 years old), education pathway (fulltime vs. dual/modular), education level (pre-university/secondary vocational vs. higher professional/university), living situation (independent vs. with parents/caregivers), informal caregiving (no vs. yes). For all analyses, a standard alpha level of 0.05 was used. Internal consistency was evaluated using Cronbach's α. Cronbach's α above 0.70 indicate positive internal consistency (Tavakol and Dennick, 2011).

Following separate analyses of the qualitative and quantitative data, the findings were integrated during interpretation. The qualitative findings were used to provide a deeper understanding of the quantitative results and to explore how both datasets related to the study's conceptual model.

### 2.4 Ethical considerations

Prior to participating in the study, students were provided with written information outlining the study’s objective, procedures and measures in place of safeguarding their privacy. Students were informed about the voluntary nature of their participation and their right to withdraw from the study at any time without needing to provide a reason. All participating nursing students provided informed consent. All interviewed students were allocated pseudonyms to protect anonymity. In accordance with the guideline of the Central Committee on Research Involving Human Subjects, this study falls outside the scope of the Dutch Medical Research Involving Human Subjects Act (WMO). The researchers have contacted the ethical committee for general advice on the ethical appropriateness of this study. No issues were reported.

## 3 Results

### 3.1 Student characteristics

For the qualitative study, 15 nursing students participated in individual interviews. Participants ranged in age from 19 to 26 years, and nine were female. Eight students were enrolled in the full-time program, and seven in the dual program. Eight participants were in their third year of study and seven in their fourth year. Five participants were progressing through the study program according to the intended study trajectory, whereas ten had experienced study delay of at least six months.

For the quantitative study, 873 eligible nursing students were approached to participate in the online survey, of which 137 (16%) responded. Student characteristics of both study components are presented in Table 1.

**Table 1:** Student characteristics of participants in the qualitative (n = 15) and quantitative (n = 137) study components.

Characteristic	N (%)	
	Qualitative study (N = 15)	Quantitative study (N = 137)
Mean age in years (SD)	23	24 (6.6)
Gender		
Female	9	119 (87)
Male	6	16 (12)
Non-binary	0	2 (1)
Education pathway		
Fulltime	8	77 (56)
Modular	0	7 (5)
Dual	7	53 (39)
Education level		
Higher general secondary or pre-university education		91 (66)
Secondary vocational		35 (26)
Higher professional		9 (7)
University		2 (1)
Living situation		
Alone		20 (15)
With partner and/or children		22 (16)
With parents/caregivers		77 (56)
With other students		18 (13)
Work situation		
Paid work (>20 h a week)		67 (49)
Paid work (<20 h a week)		52 (38)
No work		13 (10)
Unpaid/voluntary work		5 (4)
Informal caregiving		
Yes		17 (12)
No		120 (88)

### 3.2 Interview results

The narrative begins by describing student adversities and their impact, providing essential context. Next, three themes are presented that illustrate how nursing students apply self-compassion and self-leadership skills to overcome these adversities: (Kox et al., 2020) ‘Connecting with your inner nurse', (Canzan et al., 2022) ‘Being a brave nursing student', and (Cooper et al., 2020) ‘Building strength through self-compassion’. Each theme also explores personal challenges and growth in dealing with adversities. An overview of the developed themes and subthemes is shown in Table 2.

**Table 2:** Themes reflecting how nursing students develop resilience through self-compassion and self-leadership.

Theme	Definition	Subthemes
(1) Connecting with your inner nurse	Connecting the nursing role to your values and identity. Knowing your motivation and reflecting on your actions, thoughts and feelings during or after a difficult situation.	• Self-awareness and self-insight
(2) Being a brave nursing student	Acting despite possible consequences or exposure and talking to others or asking for help during difficult situations.	• Seeking support
		• Taking action in a vulnerable position
(3) Building strength through self-compassion	Being kind, positive, and considerate towards yourself during difficult situations, accepting changes and interacting with others to not feel alone in the given situation.	• Self-care practices
		• Being kind to yourself
		• Finding comfort in shared experiences

#### 3.2.1 Adversities and their impact on nursing students

Participating students started their education motivated by the prospect of earning a nursing degree or the prospect of roles as ambulance- or oncology nurses. They appreciated challenges during internships and valued positive feedback about their work. However, adversities related to an inadequate learning environment and perceived unsafe working conditions in internships challenged their study motivation.

Students described their learning environments as inadequate, citing factors such as the fast pace of care delivery at the expense of genuine patient interaction, high work pressure, and excessive responsibilities. Chris, a nursing student, shared a particularly distressing experience: “*Once I had one [patient] who almost died, and I was alone with a temporary worker… I had to deal with this on my own*.” These challenges were often exacerbated by a lack of support and supervision. Chris further explained, “*My greatest struggle was with my supervisor, the person who was supposed to coach and assess me. But that was the person I worked the least with*.” Such experiences highlight a critical tension: students felt a strong sense of responsibility but often lacked the skills or guidance needed to manage demanding situations effectively. In this context, the absence of sufficient supervision served as a missing resource, one essential for skill development and professional growth. Without it, the sense of being underprepared remained unresolved, limiting students’ opportunities to develop confidence and competence in their roles.

In addition to structural challenges, some students perceived their working conditions as socially and emotionally unsafe. This sense of unsafety often stemmed from workplace gossip about interns and dismissive or exclusionary remarks from colleagues. Sabrina described feeling alienated due to her status as a bachelor-level student working among secondary vocational trained nurses: “*They [colleagues] thought like oh, so you are a bachelor student with these vague assignments, and you know everything better.”* Such perceptions of being devalued or “othered” contributed to a sense of not belonging. Hostile or discouraging feedback further intensified these experiences, as illustrated by Laura’s account: “*And just to hear that I probably shouldn’t become a nurse.*” These remarks exemplify workplace incivility; subtle, often indirect behaviors that undermine psychological safety and collegiality. For nursing students, such incivility may erode confidence, hinder learning, and contribute to emotional distress.

These adverse circumstances imposed an emotional and cognitive burden on students. They frequently expressed feeling stressed and exhausted during their internships. Some described falling into negative mental patterns, unable to break free from recurring negative thoughts and emotions. Others reported a broader negative cycle in their daily lives, consisting only of working and sleeping, with no time to slow down, recover, or reflect on the day. Panic and distress were common, with Lotte stating: *“I think what I felt was panic and sadness”.*

These emotions were often intertwined with a fear of failure. Sabrina shared: *“It had a profound impact on me, as I felt fear of failure and stress: am I doing it right or not?”* This uncertainty triggered rumination about themselves and their studies. Laura reflected: *“What is it that they don’t like about me?”* Many contemplated quitting their internship, further intensifying their inner struggles. Julia mentioned a conversation with a supervisor who asked*, “If this is so difficult, is this the right path for you?”* She added: *“That feels like failure… I thought: if I quit, I'll face further delay in finishing my studies, and what if I stop again?”*

The themes below present strategies that students came up with for addressing adversity, guided by the principles of self-leadership and self-compassion.

##### 3.2.1.1 Connecting with your inner nurse

This theme highlights the importance of connecting with students' "inner nurse": the integration of their personal values and identity into their nursing role, as a key source of motivation. It evokes a sense of passion, joy, and a genuine interest in the nursing profession, especially in providing care for others. Through this connection, they gained a clearer understanding of why they had chosen to study nursing, which fostered a deeper sense of meaning and purpose. This autonomous motivation helped them to stay committed to continuing their studies despite setbacks. Sol shared her childhood dream: *“Since I was little, I’ve enjoyed helping people. I wanted to be close to people and help them; being a nurse allows me to do that in the best way possible”*. However, students felt demotivated when they were unable to offer patients genuine attention, as it conflicted with their core nursing values. Julia reflected: “*It started to go against my principles… I wanted to take the time to help someone".* Additionally, witnessing disrespectful behavior toward patients contributed to this demotivation, as Sabrina noted*: "The way some colleagues treated the clients, I didn’t like it. It wasn't the image I had of nursing. It really wasn't the way I wanted it to be.”*

These experiences underscore a key tension within autonomous motivation: while it can serve as a powerful driver of perseverance and engagement, it may also lead to emotional conflict when students are placed in environments that prevent them from upholding their professional ideals. This disconnection between values and practice can become a source of moral distress, highlighting the importance of supportive learning environments that enable students to act in line with their inner motivations.

###### 3.2.1.1.1 Self-awareness and self-insight

Connecting with their feelings and motivation enabled students to stay engaged and express their opinion, supporting their resilience. Through reflective activities such as journaling and self-observation, students gained insight into their behavior and coping strategies, which in turn enabled them to approach situations differently. For example, Chris paid attention to his own reactions: *“In the past, I wouldn’t have reacted that way. Now, I dare to participate in discussions in the ward and share my opinion”.* Sabrina found journaling effective for gaining insights, while Owen emphasized the importance of pausing to carefully weigh options before acting*.* These practices of self-awareness helped students strengthen their connection with their "inner nurse", which supported them to persevere through adversities.

Internships, while challenging, enhanced students' personal and professional self-awareness. Chris highlighted his growth in confidence over time, recognizing that he struggled to stand up for his learning process in his third year. He reflected*: "I believe that I have grown self-confident thanks to the internships… Now I'm in my fourth year, and I finally am where I want to be."* He credited a safe learning environment and prior internship experiences for his growth. Beyond growth in self-confidence, some students gained a deeper, more compassionate understanding of others' struggles with mental health through their own experiences. Sabrina admitted: *“I used to think that it's up to you, you are doing that yourself, it is easy to get rid of. After experiencing my own setbacks, I now understand what it means to have mental health issues."* This student moved from a place of misunderstanding, believing that mental health issues are easily resolved and within personal control, to one of empathy and awareness after personally facing adversity. These insights emphasize how reflective practice and personal challenges enriched students' inner connection with nursing.

The connection with their inner nurse cultivated a sense of purpose and self-awareness, laying a foundation for the courage and problem solving needed to withstand the pressures of nursing education.

##### 3.2.1.2 Being a brave nursing student

This theme explores the proactive efforts students make to improve their situation, often demonstrating courage in the process. Two subthemes were found: seeking support and taking action in a vulnerable position.

###### 3.2.1.2.1 Seeking support

Students often sought support from family and friends by talking or asking for help to feel better. When necessary, students turned to professional mental health services. However, seeking professional support was a significant barrier for students as it required considerable courage to acknowledge their mental struggles and to confront the lingering stigma surrounding mental health support. Julia recounted: *“At some point it didn’t go that well. That was the moment I decided to see a psychologist”.* Seeking professional help reflected that Julia acknowledged she could not do it alone and her courage in addressing mental concerns.

###### 3.2.1.2.2 Taking action in a vulnerable position

Students took courageous steps, despite feeling vulnerable in the hierarchical power dynamics between students and nursing staff who could pass or fail their study assessments. They took action by speaking up and addressing concerns with colleagues, confronting supervisors, or presenting themselves as equals, actions that had a risk of rejection. These experiences often made them feel exposed or uncertain. Ed exemplified this when he openly discussed negative feedback from his supervisor: *"While I was working with my supervisor to assist a patient, she started criticizing me in the patient's presence, insisting that my approach was incorrect and explaining how differently I should be doing it. […] You could see the patient's discomfort. I decided to leave the room and wait until I had cooled down. Later that day, I discussed the incident with my supervisor, explaining the impact of her behavior on me."* Speaking up was a difficult but necessary step for Ed to regain a sense of control over the situation. Other students showed assertiveness by addressing concerns with supervisors. Sabrina, for example, recounted a confronting conversation: *“I really had to be strong and gather a lot of courage to have that conversation. Because… I was sitting across from my supervisor saying: you know, you don’t meet my expectations. I said it, even though it was very hard for me, because I felt insecure and vulnerable.”* This action empowered Sabrina to make the changes needed to get out of an inadequate learning environment that failed to support her professional growth. Some students decided to invest in building relationships with their supervisor and actively responded to their feedback. They showed braveness by making the effort to connect with someone in a different hierarchical position and by positioning themselves as equals. Lotte shared: *“I really put effort into the relationship with my supervisor."*

Many students struggled with being courageous. To please others, they sometimes decided not to speak up or set boundaries. For instance, Chris and Julia sacrificed study hours to assist colleagues. Other students expressed hesitance to show vulnerability by sharing personal experiences with classmates, fearing they might be judged. Sol shared: *"I found it difficult, because you have to admit it, and people might not understand. They might think: ‘things happen to me too.’ Then I think: what's the point of telling them? They're just my classmates, they don't need to know."* In contrast to showing his true self, Owen chose to adapt his behavior to meet his supervisor’s expectations: *“As long as I adjust a bit to her way… She is more serious [than me] at work… So, when working with her, I adjust to her.”* This strategic approach was aimed at securing a good grade for his internship, exemplifying the pressure students can feel to perform rather than showing their true personality: *"A bit nonchalant, a bit... I don't want to give the patients the impression that I'm too strict. I want them to feel free to say something serious, and for me to be someone who can really bring that to the doctor… Someone who stands between the doctor and the patient, that's how I try to position myself. I try not to present myself like a doctor; that feels too impersonal, you know? And I get the sense that she is much more like that. I do have to adjust to that, watch what I say sometimes."*

Through these adversities, students developed greater confidence and learned to advocate for themselves. They began addressing problems earlier during subsequent internships by providing feedback, being more open with colleagues about their thoughts and emotions and proactively identifying what they wanted to learn instead of waiting passively. Chris reflected on his growth: *“I’ll actively look for opportunities myself instead of just waiting for something to happen… Now I can say what I need or wish to do and learn.”* Similarly, Ed highlighted his development in giving constructive feedback in challenging situations: *"The only thing I would do differently is the way I give feedback, especially to a colleague who is so different from me… I just want to share my perspective and avoid accusations*.” These reflections demonstrate his growth in expressing himself in a respectful and constructive manner, even to someone who may be perceived as holding a higher status compared to an intern.

Seeking support and taking action in a vulnerable position have shown to be difficult actions that reflected the students' bravery. To remain courageous and resilient, many found it helpful to apply self-compassion strategies.

##### 3.2.1.3 Building strength through self-compassion

Self-compassion supports students to overcome adversities and recover from their impact. They reported that by practicing self-care, treating themselves with kindness, and connecting with peers facing similar challenges, they were able to regain strength.

###### 3.2.1.3.1 Self-care practices

Regardless of the type of leisure activity, several students highlighted the importance of actively setting aside time for self-care to recharge, even amidst their demanding work and study schedule. They mentioned a variety of creative, relaxing, and physical activities, some of which included friends, while others involved me-time in solitude. For instance, Sol mentioned: "*I hike a lot… I try to use my phone less, go outside and just sit and read… Things like that help me take better care of myself”*. Moreover, Jennifer commented: *"I could stop thinking of the internship and enjoy my time outside of it. I make sure to maintain a busy social life, so after a difficult day, I can easily let go.”* Facing adversity fostered growth in recognizing the importance of maintaining a good work-life balance. Sabrina shared: *“It is important to enjoy your time as a student… Do fun things too… That is what I’ve learned.”*

###### 3.2.1.3.2 Being kind to yourself

As part of self-compassion, students practiced being kind to themselves. They adopted an accepting attitude toward their situation. For some students, this meant deciding to quit their internship to protect themselves, even though it meant accepting study delays or that their educational path differed from others. Julia's words illustrate this: *"The question was whether I would make it or not. The options were [to stop the internship and] face a six-month delay or [to continue and] endure six months of stress and still face the delay. I decided to stop, do fun things, work on myself for six months, and make a fresh start elsewhere. So, I had six months of rest*.” Positive words and lowering expectations helped students recover from difficult situations during their internships and better cope with future challenges. Sometimes, it helps to simply let go of setbacks and accept that not everything will go as planned, as Laura expressed how reflection helped her to be less rigid about her expectations: *“Nowadays I think I'll just do it, I'll try. And if it doesn’t work out, then it doesn't work out. Tomorrow is a new day*.” Another way students showed kindness to themselves was by cultivating a positive mindset. For instance, Chris expressed pride in how he handled a critical acute situation on his own, without a supervisor: *"You doubt yourself a lot when you have to act in an acute situation - am I doing this right? Am I following the protocol? Normally, you check with your supervisor for confirmation, but there wasn't one. Later, I looked back and saw how I acted quickly and correctly at that moment. I could give myself a pat on the back*.”

###### 3.2.1.3.3 Finding comfort in shared experiences

Moreover, an important aspect of self-compassion was finding comfort in shared experiences. Students reminded themselves that they were not alone. Sabrina, for instance, expressed dissatisfaction with her internship and the supervision she received. Initially, she felt isolated in this experience, but that changed when she shared her situation with another student who was going through something similar. Sabrina reflected: *“She [the other student] experienced the internship the same way I did… It was so nice talking to her about it and sharing our problems. Otherwise, I would have felt really lonely.”* Chris also mentioned how bonding with fellow interns led to lasting friendships.

Nevertheless, some students found it challenging to practice self-compassion. Several still felt isolated in their experiences, as Laura shared: *“I really thought this only happens to me.”* Others struggled to maintain a healthy work-life balance amidst the demands of work and school. Ed expressed a desire to spend more time relaxing: *“Now I spend a lot of time working and studying… and recuperating from it*.” High expectations have also proved difficult for some students. Sabrina reflected: *“With some assignments, I start doubting, setting the bar too high.”* while Laura admitted to being self-critical: *“Like ‘you can’t do it, you are a failure’...”*

### 3.3 Survey results

The self-compassion mean total score (range 1–7) was 4.11 (α = .85; SD = 0.92), the vitality mean total score (range 1–7) was 4.50 (α = .90; SD = 0.92), and the self-leadership mean total score (range 1–5) was 3.38 (α = .74; SD = 0.60). Pearson correlation analyses between vitality, self-leadership, and self-compassion revealed all correlations <.70 (see also the correlation matrix in Supplementary Files II).

A hierarchical multiple regression analysis was conducted to examine whether self-compassion and self-leadership predicted vitality while controlling for sociodemographic characteristics. In Step 1, the sociodemographic factors were entered as control variables. This model explained 9.2% of the variance in vitality, but was not statistically significant (*R²* = .092, *p* = .240). The addition of self-compassion in step 2 significantly improved the model, accounting for an additional 27.6% of the variance in vitality (Δ*R²* = .276, *p* < .001). In Step 3, self-leadership explained an additional 17.2% of the variance in vitality (Δ*R²* = .172, *p* < .001). The final model explained 54% of the variance in vitality (*R²* = .540; adjusted R^2^ = .495).

These findings indicate that self-compassion and self-leadership make substantial contributions to vitality. Self-compassion accounted for the largest increase in explained variance (27.6%), whereas self-leadership explained an additional 17.2%, suggesting that both constructs are important predictors of vitality among nursing students. The results of the hierarchical regression analysis are presented in Table 3.

**Table 3:** Hierarchical regression analysis predicting vitality among nursing students.

Variable	Model 1 β	Model 2 β	Model 3 β
Gender	.133	.062	.014
Age	.078	.022	.039
Year of study	-.009	-.021	.005
Educational pathway	.098	.112	.092
Educational level	.128	.069	.040
Living situation	.007	-.007	.049
Work situation	-.042	-.046	-.037
Informal caregiving	.042	.082	-.012
Self-compassion	–	.542^a^	.482^a^
Self-leadership	–	–	.435^a^
R²	.092	.368	.540
Adjusted R²	.023	.313	.495
ΔR²	.092	.276^a^	.172^a^
F	1.324	6.676^a^	11.997^a^
ΔF	1.324	45.013^a^	38.186^a^

a *p* < .001.

## 4 Discussion

This study investigated the role of self-compassion and self-leadership skills in building resilience among nursing students. Results show that nursing students faced adverse circumstances during their internships and demonstrated resilience in response to inadequate learning environments. These environments were often characterized by a lack of supervision, creating a tension between students' strong sense of responsibility and their limited competence and support to manage demanding situations effectively. Additionally, students persevered in socially and emotionally unsafe working conditions, including subtle exclusion or offensive remarks directed at student interns. These students are in a transitional phase of their career development, because they experience extended immersion in clinical practice rather than classroom-based learning for the first time. This phase is unique and increases both learning demands, emotional demands, workplace demands, cognitive load and a lack of personal resources (e.g., occupational efficacy) that might otherwise be protective.

Quantitative findings revealed that both self-compassion and self-leadership were significant predictors of vitality. Self-compassion explained the largest proportion of variance in vitality, whereas self-leadership also made a substantial contribution. These findings suggest that both a compassionate attitude towards oneself and the ability to proactively regulate one's thoughts and behaviors are important resources for adaptive functioning during clinical placements in Dutch nursing students. The qualitative findings help explain these quantitative relationships by illustrating how students applied self-compassion through self-care and self-kindness, and self-leadership through reflective practice, help-seeking, and courageous action in challenging situations.

These results provide evidence for incorporating leadership and self-compassion training into nursing education, especially during internships. The qualitative findings suggest that these capacities support resilience processes, while the quantitative findings indicate that they are associated with higher vitality among nursing students. This aligns with previous research on their importance on building resilience among nursing students (Kotera et al., 2021; Seo and Ko, 2024). The qualitative findings describe how nursing students navigate difficult situations through courageous actions, connecting with their 'inner nurse', and self-compassion practices. These skills are necessary to withstand difficult working circumstances including a heavy workload, limited support, and hierarchical cultures, which are also reported as reasons for leaving the nursing profession (Bahlman-van Ooijen et al., 2023). The current study extends this knowledge by demonstrating that students face an additional challenge due to their subordinate, dependent, and temporary roles within nursing teams during internships. Even before nurses enter the workforce, they are confronted with these circumstances, contributing to a negative perception of the nursing profession and increasing turnover. It is therefore crucial to adopt a holistic approach to resilience that acknowledges the unique challenges faced by nursing students. Healthcare organizations and educational institutions must work collaboratively to create a welcoming environment for students. For example, social support, psychological safety, work-life balance and sense of belonging contribute to nursing students’ resilience (Hughes et al., 2022). Therefore, providing systemic support should be a priority, rather than placing the responsibility for resilience solely on individual students. This aligns with Traynor's critical perspective on resilience, which challenges the idea that students must adapt individually to structurally adverse circumstances (Traynor, 2017).

An existing concept analysis of nurse resilience found key attributes including social support, self-efficacy, work-life balance, humor, optimism, and being realistic (Cooper et al., 2020). Results from our current study complement this framework with factors that are crucial specifically for nursing students. Connecting with one’s inner nurse emphasizes the importance of forming a professional identity in sustaining motivation during adversity. Being brave reflects the courage required to seek support, speak up, and take action despite students’ dependent and vulnerable position within clinical hierarchies. Building strength through self-compassion emphasizes the importance of responding to setbacks with self-compassion.

Although self-compassion practices were applied to build resilience, it was evident that some students felt reluctant to let their guard down in the classroom and share their personal struggles. On the one hand, this could be related to perceived psychological safety and existing norms in the classroom. In addition, fear of compassion might play a role (Gilbert et al., 2011). Nursing students might feel anxious about practicing self-compassion by not allowing themselves to receive the support and care they need from their peers. Alternatively, they might be frightened to receive compassion from others, which they respond to by putting up barriers or keeping their distance. This aligns with a recent study among nursing students, which suggests that reducing fear of compassion is an important step toward improving emotional intelligence and compassion competence (Sadeghi et al., 2025).

### 4.1 Practical implications

Strengthening nursing leadership strategies in education, like taking action while being conscious about your dependent role, can empower students to respond to adversities more successfully. Moreover, encouraging a culture of self-compassion in the clinical and educational environment enables students to decrease stress resulting from adverse circumstances. These interventions may improve retention as students step into professional roles during internships.

This study focused on individual internal strategies nursing students can develop to become and remain resilient. Educational and clinical nursing institutions could invest in training that fosters individual nurse resilience. At the same time, it is important to incorporate a holistic, systemic approach. This requires initiatives to support nursing students' wellbeing and resilience, such as providing appropriate levels of responsibility, offering adequate support, and cultivating a welcoming and inclusive culture. Moreover, nursing leadership strategies should focus on improving shared governance, empowering (future) nurses by breaking down hierarchical structures, and ensuring their voices are heard (Paine and Prochnow, 2022).

Educational institutions could invest in mindfulness techniques; stable, year-long mentorship; and stimulate honest and vulnerable dialogue among peers in reflective sessions. Simulation exercises can be a practical tool to help students anticipate and navigate setbacks during internships. To cultivate a supportive organizational culture, acceptance and understanding from colleagues and supervisors is crucial. Positive feedback and genuine interest from staff are significant motivators. Collaborative relationships and regular feedback sessions, including daily evaluations, can be effective practices for fostering a sense of belonging and continuous professional improvement.

### 4.2 Limitations

First, this study was conducted within a single educational institution, which may limit the generalizability of the findings to other educational contexts. Second, a general vitality instrument with a resilience subscale was used rather than a specific resilience measure. Although the Vita-16 includes a resilience subscale, the factor structure in our sample did not support separate analysis of this subscale. Therefore, only the total vitality score was used and reported. As a result, the quantitative findings should be interpreted as reflecting vitality rather than resilience specifically.

Third, the qualitative sample was partly recruited through convenience sampling, which may have introduced selection bias. And finally, the relatively low response rate (16%) of the quantitative study component may have introduced non-response bias. Students who participated may have differed from non-participants in terms of motivation, well-being, or internship experiences. Therefore, the findings should be interpreted with caution, as their generalizability to the wider nursing student population may be limited.

### 4.3 Suggestions for future research

Future studies could investigate the feasibility and efficacy of interventions aimed at fostering resilience through self-compassion and self-leadership. Promising approaches include self-compassion interventions and resilience training programs that are cost-effective, time-efficient, and adaptable to diverse settings and populations. Evaluating their impact on nursing students’ resilience would provide valuable insights into effective educational strategies for building a more resilient nursing workforce. Moreover, it is recommended to investigate resilience through a critical, systemic lens, emphasizing the shared responsibility to support and safeguard the well-being of the (future) nursing workforce.

## Ethics declaration

Written informed consent to take part in the study and to publish the article has been obtained from all participants or their legal representatives. The privacy rights of participants have been observed.

This study was performed in compliance with relevant laws, regulatory frameworks and guidelines where the research took place. This study was conducted in accordance with WMO (Dutch). Ethics committee approval was not required under relevant laws and institutional guidelines. In accordance with the guideline of the Central Committee on Research Involving Human Subjects, this study falls outside the scope of the Dutch Medical Research Involving Human Subjects Act (WMO).

## Funding

This research did not receive any specific grant from funding agencies in the public, commercial, or not-for-profit sectors.

## Study registration

Not registered.

## CRediT authorship contribution statement

**Heleen C. Melissant:** Writing – review & editing, Writing – original draft, Visualization, Supervision, Project administration, Methodology, Investigation, Formal analysis, Conceptualization. **Claudia M. Hernandez Murillo:** Writing – review & editing, Writing – original draft, Project administration, Methodology, Investigation, Formal analysis, Conceptualization. **Kerry A. Sherman:** Writing – review & editing, Supervision, Methodology, Formal analysis, Conceptualization. **Monique F. Crane:** Writing – review & editing, Supervision, Methodology, Formal analysis, Conceptualization. **Rick R.A. Hendriks:** Methodology, Formal analysis. **Kim J. Verhaegh:** Writing – review & editing, Supervision, Resources, Funding acquisition, Formal analysis, Conceptualization.

## Declaration of competing interest

The authors declare that they have no known competing financial interests or personal relationships that could have appeared to influence the work reported in this paper.

## Data Availability

Survey respondents were assured in the informed consent form that raw data would remain confidential and would not be shared. Data not available / The data that has been used is confidential.
