# Supplementary material for: How nursing students develop resilience through self-compassion and self-leadership: a mixed methods study
Source: Int J Nurs Stud Adv. 2026 Oct 1;11:100693. doi: 10.1016/j.ijnsa.2026.100693 (PMC13676078; doi:10.1016/j.ijnsa.2026.100693)
Supplement: Supplementary file 1 [file mmc1.docx]

**SUPPLEMENTARY FILE I**

Topic list exploring resilience, self-compassion and self-leadership in nursing students.

| 1. What do you like about nursing? 2. Why did you want to become a nurse? | |
| --- | --- |
| 3. What gives you joy and energy during your studies? | |
| 4. What are you proud of, and what are your loved ones, colleagues, or fellow students proud of in you? | |
| 5. Tell me about your most recent internship. | - Think of a situation where you experienced pain, stress, humiliation, feelings of failure, rejection, or harsh criticism. Or a situation that drained your energy or left you feeling dissatisfied. |
| 6. How consciously did you experience that moment? | - What helped you or prevented you from being aware of it? - What emotions did you feel? - To what extent were you able to view the situation from a distance and reflect on your response? |
| 7. How did you feel at that moment? | - Could anyone have experienced a similar situation? - What thoughts did you have about the situation, yourself, and your physical health? |
| 8. How did you handle that difficult situation, or how did you approach it? | - Did you try to resolve it, avoid it, seek support, feel helpless, express your feelings, or use reassuring thoughts? - What exactly did you do? - How did you manage to distract yourself or shift your thoughts? - How did you feel about making choices during that time? - How did you respond to suggestions or expectations from others? - What actions did you take to change the situation? - Did you set specific goals for yourself? - Did you visualize or mentally rehearse for challenging situations? How did that go? |
| 9. How do you relate to yourself during this situation? | - What did you say to yourself? - How much did you need encouragement, understanding, patience, empathy and kindness? - To what extent were you able to provide these for yourself? - What was your image of yourself at that moment? |
| 10. What gave you the strength to endure that challenging situation? | - What did you do to stay resilient? - How did faith or sense of meaning/purpose contribute to this? - What role did goal setting and motivation play in your life or studies during that time? |
| 11. What support did you receive from work or your internship during this situation? | - How did direct colleagues respond to you and your situation? - Did you feel sufficiently supported? In what ways? - And how about team leaders and supervisors? - How did this support influence your ability to take care of yourself and recover? - What did you truly need from your work environment at that time? From whom did you seek or receive support? - Who could you genuinely talk to? - Did you experience the learning work environment as safe? |
| 12. What support did you receive from school during this situation? | - How did the internship office and lecturers respond to your situation and support you? - What was the response from fellow students? - What form did this support take? - What did you truly need from school, or lecturers at that time? - Did you feel comfortable being open during lessons? - Could you share what really affected you and admit what you find difficult? - What made it challenging to do so? |
| 13. How quickly did you recover after that difficult time? | - How quickly did you adjust to the new or changed situation? - How did you recover from this event? - For example, how do you set boundaries and create space for your own needs? - How did you learn to do this? |
| 14. Looking back, what would you have done differently? | - What did you learn from it? - What would you have liked to have done differently? - What did you miss? - What would you recommend to others? - What valuable lesson did you learn from the experience? - How do you look back on it now? - What would you do the same or differently if it happened again? |
| 15. How could the school or lecturers better support you in learning to deal with setbacks, difficult, stressful, or painful situations? | - What do you think you need to cope with challenging situations in your future work? - How could your studies help with this? - What support did you miss from the internship office, lecturers, or others at school during such times? |
