# Supplementary material for: How nursing students develop resilience through self-compassion and self-leadership: a mixed methods study
Source: Int J Nurs Stud Adv. 2026 Oct 1;11:100693. doi: 10.1016/j.ijnsa.2026.100693 (PMC13676078; doi:10.1016/j.ijnsa.2026.100693)
Supplement: Supplementary file 2 [file mmc2.docx]

**SUPPLEMENTARY FILE II**

Correlation matrix with means and SD's for self-compassion, self-leadership and vitality.

| Variable | M | SD | Self-Compassion | Self-Leadership | Vitality |
| --- | --- | --- | --- | --- | --- |
| Self-Compassion (α = .85) | 4.11 | 0.92 | — | 0.15 | 0.57** |
| Self-Leadership (α = .74) | 3.38 | 0.6 | 0.15 | — | 0.52** |
| Vitality (α = .90) | 4.50 | 0.92 | 0.57** | 0.52** | — |

** Correlation is significant at the 0.01 level (two-tailed).
